# Is oxidative stress evaluated in viable human spermatozoa a marker of good semen quality?

**DOI:** 10.3389/fendo.2022.1012416

**Published:** 2022-11-22

**Authors:** Giulia Traini, Lara Tamburrino, Linda Vignozzi, Elisabetta Baldi, Sara Marchiani

**Affiliations:** ^1^ Department of Experimental and Clinical Biomedical Sciences “Mario Serio”, University of Florence, Florence, Italy; ^2^ Department of Experimental and Clinical Medicine, University of Florence, Florence, Italy; ^3^ Andrology, Women’s Endocrinology and Gender Incongruence Unit, Careggi University Hospital, Florence, Italy

**Keywords:** human spermatozoa, reactive oxygen species, CellROX^®^ Orange Reagent, dihydroethidium, sperm motility, sperm DNA fragmentation, hyperactivation, apoptosis

## Abstract

**Background:**

Oxidative stress is defined as the unbalance between reactive oxygen species (ROS) production and antioxidant defences. Whereas low levels of ROS are necessary for physiological sperm functions, high levels impair fertility damaging membranes, proteins and DNA. In this study, we used two probes, CellROX^®^ Orange and Dihydroethidium (DHE), which reveal different intracellular ROS species, to evaluate the association between the percentage of oxidized viable spermatozoa and sperm functions.

**Methods:**

The percentage of oxidized spermatozoa was evaluated by flow cytometry with the two probes concomitantly with standard semen parameters and sperm DNA fragmentation (sDF, by TUNEL/PI). Phosphatidylserine membrane exposure, caspase 3,7 activity, sperm kinematic parameters and hyperactivated motility were evaluated by Annexin V, FLICA™ and CASA system respectively.

**Results:**

Oxidized viable spermatozoa, evaluated with both probes, were positively associated with sperm basal parameters and negatively with sDF. Also, we found that a consistent percentage of CellROX^®^ positive viable spermatozoa were selected from whole semen during swim up procedure. Double staining of CellROX^®^ Orange with Annexin V and FLICA™ demonstrated that viable oxidized spermatozoa do not show apoptotic features.

**Conclusion:**

Overall, our results suggest that CellROX^®^ Orange and DHE allows identification of the viable oxidized sperm fraction related to better performances.

## Introduction

Approximately fifty percent of couple infertility cases can be attributed to male factor, alone or in combination with the female one (1). Oxidative stress, characterized by an imbalance of reactive oxygen species (ROS) and antioxidant defences, is considered one of the causes of idiopathic male infertility (2, 3). ROS are free radicals derived from oxygen with unpaired electron that makes them very reactive, and include superoxide anions (O2^•-^), hydrogen peroxide (H_2_O_2_), peroxyl (·ROO) and hydroxyl (·OH) (2, 3). Several may be the sources of ROS in semen, both endogenous and exogenous. Spermatozoa and leukocytes are the main endogenous sources of ROS, whereas exogenous causes include unhealthy lifestyle, smoking, alcohol intake, radiations, pathological conditions and environmental pollutants (4, 5).

In physiological conditions, seminal ROS are balanced by antioxidant activities, which likely maintain ROS at the low levels required for the main sperm functions, such as motility, capacitation, hyperactivation (6) and acrosome reaction (7). However, when ROS are overproduced or poorly counteracted by antioxidant activities, they damage membranes (8), proteins (9) and DNA (4), resulting in an impairment of sperm functions. In particular, at DNA level, ROS may produce base oxidation, mutations and fragmentation (4).

Evaluation of seminal or sperm oxidative status could add more information regarding male fertility potential to that obtained from routine semen analysis, especially in particular medical conditions, such as inflammatory statuses of the male genital tract, where cytokines or other substances that can stimulate ROS production by spermatozoa or other cells present in the ejaculate are produced (10). Over the years, different methods have been developed to measure ROS in semen (11). Some of these assays measure ROS levels, which have the limitation of detecting total semen ROS without distinguishing among the various species, or evaluate semen oxidation-reduction potential. Moreover, these assays are not able to detect if ROS are produced by spermatozoa or other cells present in the ejaculate (11). Alternatively, commercial fluorescent probes can be used, which have the advantages of evaluating intracellular ROS and to be more specific towards the different ROS species, but cohort studies for validation of these methods in human semen are lacking.

Recently, Escada-Rebelo et al. (12) compared several commercially available fluorescent probes to define their distinct specificities to detect ROS and reactive nitrogen species in human spermatozoa. Among these probes, CellROX^®^ reagents appear interesting as they detect the oxidative status in viable spermatozoa. A viable oxidized spermatozoon, if preserving motility, might participate in the fertilization process *in vivo* and during *in vitro* fertilization (IVF) and intracytoplasmic sperm injection (ICSI) procedures. However, whether oxidized viable spermatozoa retain functions and other features important for male fertility potential is presently unknown.

According to supplier’s datasheet, CellROX^®^ cell-permeant reagents are non-fluorescent in a reduced state, but emit a strong fluorescent signal when reacting with ROS in live cells. The fluorescent signal can be then detected both by fluorescent microscopy and flow-cytometry. In particular, the study of Escada-Rebelo et al. (12) concluded that CellROX^®^ Orange, among the various CellROX^®^ reagents, shows a specificity for detection of H_2_O_2_. Another interesting fluorescent probe is Dihydroethidium (DHE), which detects H_2_O_2_ and O2^•-^ species both in viable and unviable spermatozoa (12).

In this study, we used CellROX^®^ Orange and DHE coupled to flow cytometry to evaluate the association between the percentage of viable (with both probes) and unviable (with DHE) oxidized human spermatozoa and routine semen parameters and sperm DNA fragmentation (sDF). In addition, we evaluated whether viable oxidized spermatozoa are selected during swim-up procedure, which is used to select spermatozoa for assisted reproduction. Finally, we evaluated the concomitant presence of ROS and the apoptotic markers Annexin V and caspase 3, 7 in viable spermatozoa.

## Material and methods

### Chemicals

Human tubal fluid (HTF) medium and human serum albumin (HSA) were purchased from Fujifilm Italia S.p.A. (Milan, Italy). CellROX^®^ Orange Reagent, LIVE/DEAD™ Fixable Green Dead Cell Stain (L23101), Vybrant™ FAM Caspase -3 and -7 Assay Kit and Annexin V Alexa Fluor^®^ 488 conjugate were purchased from Invitrogen by Thermo Fisher Scientific (Waltham, MA, USA). *In Situ* Cell Death Detection Kit were purchased from Roche Molecular Biochemicals (Milan, Italy). Menadione was purchased from Merck Life Science S.r.l. (Milan, Italy) and Tert-Butyl hydroperoxide was purchased from Fisher Scientific (Italy).

### Human semen samples collection and processing

The study was approved by the local ethical committee (20908_bio). Semen samples were consecutively obtained by masturbation after a minimum of two and a maximum of seven days of sexual abstinence from patients undergoing routine semen analysis for couple infertility in the Andrology Laboratory of the University of Florence. The only inclusion criterion was the attainment of signed informed consent by the patient. After initial evaluation of the samples, those from azoospermic and severe oligozoospermic subjects (< 5 million spermatozoa/ejaculate) and those with leukocytes were discarded. Semen analysis was carried out according to World Health Organization manual [V edition, WHO, 2010 (13)]. After 30 minutes from the semen collection, the volume, viscosity and pH were evaluated together with sperm concentration, progressive and non-progressive motility, total number of spermatozoa and morphology. Semen characteristics of the 165 samples used for the study are reported in **Table 1**.

**Table 1:** Median, [IQR] and (range) of semen parameters are reported in subjects (n=165) where CellROX^®^ Orange and DHE were detected.

Sample	Age (years)	Abstinence (days)	Progressive motility (%)	Total motility (%)	Concentration (x10^6^/mL)	Number (x10^6^/ejaculate)	Normal morphology (%)
n=165	37 [33-42] (range:21-61)	4 [3-5] (range:1-15)	50 [42-57] (range:8-84)	57 [49–65] (range:21-85)	48.5 [22.2–101.0] (range:2.0-352)	166.5 [77.9–327.1] (range:5.6-1228.5)	4 [2-6] (range:0-13)

For experiments evaluating the percentage of viable oxidized spermatozoa after swim up, each semen sample was divided into four experimental points: 1. whole semen at time zero (t0); 2. direct swim-up; 3. resuspended samples where, after incubation as for swim-up selection, the upper and lower media were mixed together; 4. whole semen after 45 minutes of incubation at 37°C (t45). Direct swim up selection (13) was performed in experimental points 2 and 3, by layering 1 ml HTF−10% HSA to 1 ml of whole semen and by incubating at 37°C for 45 minutes. Then, 800 μL of the upper medium phase, containing the motile fraction of spermatozoa, was collected in swim up selected samples. Sperm number and motility were evaluated and only those samples with a progressive motility >90% were used. Kinematics parameters and CellROX^®^ Orange positivity were then evaluated in all experimental points.

In some experiments, before labelling with CellROX^®^ Orange or DHE, washed spermatozoa were incubated with two inducers of ROS, Tert-Butyl hydroperoxide (TBHP, 1mM) and Menadione (12.5 and 50 µM) for 30 minutes at 37°C, 5% CO_2_ and 30 minutes at room temperature, respectively.

### Assessment of intracellular ROS

Intracellular ROS were detected by using two different probes, CellROX^®^ Orange and DHE.

For both staining, 3 x 10^6^ washed (with HTF-HSA 10%) spermatozoa were divided into two equal aliquots. One aliquot was incubated in 200 µL of HTF with CellROX^®^ Orange (1 µM) for 30 minutes at 37°C and 5% CO_2_ or DHE (1.25 µM) for 20 minutes at room temperature. For the negative control, the other aliquot was incubated with only medium for the same time and temperature of respective test samples. After incubation, three washes with PBS were carried out and the samples were resuspended in 300 µL of PBS and Yopro-1 (Y1, 25nM) was added for acquisition by flow cytometry (see below). To verify the localization of the signal related to samples stained with CellROX^®^ Orange and Y1 as well as with DHE and Y1, spermatozoa were layered on a slide and observed under Axiolab A1 FL fluorescence microscope (Carl Zeiss, Jena, Germany), equipped with Filter set 15 and 44 by an oil immersion 100× magnification objective. Some samples, before CellROX^®^ Orange labelling, were stained with the probe L23101 (diluted 1:10000 in 500 µL of PBS) for 1 hour at 37°C.

For swim up selected spermatozoa, the assessment of intracellular ROS with CellROX^®^ Orange was carried out as described above for washed semen samples.

### Assessment of sperm DNA fragmentation

sDF was detected by TUNEL (terminal deoxynucleotidyl transferase (TdT)-mediated FITC-dUTP nick end labelling) assay by using *In Situ* Cell Death Detection Kit (Roche Molecular Biochemicals, Milan, Italy) (14). Briefly, fixed spermatozoa (10 x 10^6^) were centrifuged at 500 x g for 5 minutes and washed twice with 200 µL of PBS with 1% BSA. After permeabilization in 0.1%Triton X-100 in 100 µL of 0.1% sodium citrate for 4 minutes in ice, the samples were divided into two aliquots for labelling reaction. Test sample was incubated in 50 µL of labelling solution (supplied with the kit) with the TdT enzyme for 1 hour at 37°C in the dark. The negative control was prepared by omitting TdT. Finally, samples were washed twice, resuspended in 300 µL of PBS, stained with 10 µL of Propidium Iodide (PI, 30 mg/mL), and then analyzed by flow cytometer (see below).

### Double staining with Annexin V Alexa Fluor™ 488 conjugate and CellROX^®^ Orange Reagent

A double staining with Annexin V Alexa Fluor™ 488 conjugate (used to identify early apoptotic cells) and CellROX^®^ Orange Reagent was performed and acquired by flow cytometry. 8 x 10^6^ spermatozoa were washed and resuspended in 400 μL of HTF-10%HSA added with 2 mM CaCl_2_ and then divided into two aliquots. One aliquot was incubated with Annexin V (supplied at the 100X concentration) and CellROX^®^ Orange (1µM) for 30 minutes in the dark at 37°C in a 5% CO_2_. The other aliquot (negative control) was incubated in 200 µL of HTF-10%HSA with 2 mM CaCl_2_. For flow cytometry compensation, a sample stained only with Annexin V and a sample stained only with CellROX^®^ Orange were also prepared. The samples were then acquired by flow cytometry (see below).

### Double staining with FLICA™ and CellROX^®^ Orange Reagent

Caspases activity was evaluated by using Vybrant™ FAM Caspase-3 and -7 Assay Kit based on a fluorescent inhibitor of caspases (FLICA™) according to Marchiani et al. (15). 12 x 10^6^ spermatozoa were washed in PBS and then divided into four aliquots. An aliquot was resuspended in 300 μL of PBS and added with 10 μL of 30X FLICA working solution for 1 hour at 37°C. After 30 minutes, CellROX^®^ Orange probe (1µM) was added. The negative control was incubated only with PBS. After incubation, samples were washed two times with PBS and, finally, resuspended in 300 μL of PBS and acquired by flow cytometry (see below). An aliquot incubated only with FLICA™ and an aliquot incubated only with CellROX^®^ Orange were also prepared for flow cytometry compensation.

### Flow cytometry

8000 events in the characteristic forward scatter/side scatter region of spermatozoa were acquired (14) by a FACScan flow cytometer equipped with a 15-mW argon-ion laser for excitation and analyzed by CellQuest-Pro software program (Becton–Dickinson). FL-1 (515–555-nm wavelength band) and FL-2 (563–607-nm wavelength band) detectors revealed green fluorescence of L23101, TUNEL, Annexin V, FLICA™ and Y1 and red fluorescence of CellROX^®^ Orange, DHE and PI respectively.

In the dot plot of fluorescence distribution of the negative controls, a marker, including 99% of total events, was established and then translated in the corresponding test sample. All the events beyond the marker were considered positive.

**Figure 1:**
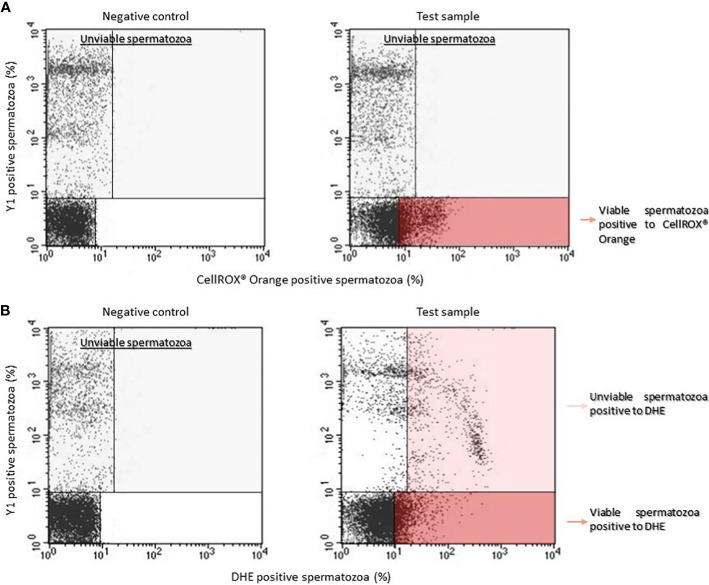
Typical dot plots representing negative control (left) and test sample (right) related to CellROX^®^ Orange **(A)** or DHE **(B)** and Y1 (ordinate, green fluorescence) stained spermatozoa. Light grey rectangles contain dead spermatozoa (events positive to Y1) whereas dark red rectangles include the percentage of viable spermatozoa positive to CellROX^®^ Orange or DHE. Note that DHE stains also unviable spermatozoa (light red rectangle).

Dot plots for CellROX^®^ Orange (panel A) and DHE (panel B) are shown in **Figure 1**. Left panels report the typical dot plots of fluorescence related to negative controls and right panels show test samples. CellROX^®^ Orange labels only Y1 negative events (viable spermatozoa in lower right quadrant). DHE labels both Y1 negative (viable sperm in lower right quadrant) and positive (upper right quadrant) events.

sDF (TUNEL-labeled spermatozoa) was determined within PI positive events of the characteristic forward scatter/side scatter region of spermatozoa (14). The percentage of TUNEL positive spermatozoa was calculated within the PI brighter population (containing both viable and unviable spermatozoa as well as both DNA fragmented and not DNA fragmented spermatozoa), the PI dimmer population (containing unviable and DNA fragmented spermatozoa) and in both sperm populations (total sDF) (14).

### Assessment of kinematic parameters and hyperactivation

To evaluate kinematic parameters and hyperactivation, the samples were analyzed by Computer-Assisted-Sperm Analysis (C.A.S.A., Hamilton Thorne Research, Beverly, MA, USA). The settings used during C.A.S.A. procedures were: analysis duration of 1s (30 frames); maximum and minimum head size, 50 and 5 µm^2^; minimum head brightness, 170; minimum tail brightness, 70. Average path velocity (VAP, µm/s), straight line velocity (VSL, µm/s), curvilinear velocity (VCL, µm/s), amplitude of lateral head displacement (ALH, µm), beat cross frequency (BCF, Hz), straightness (STR, %) and linearity of progression (LIN, %) were recorded. A fraction representing the hyperactivated spermatozoa percentage (HA, %) was identified setting manually the following threshold values: VCL ≥150µm/s, ALH ≥7µm and LIN ≤50% (16). A minimum of 200 motile cells and 5 fields were analyzed for each aliquot.

### Statistical analysis

All statistical analyses were performed using the Statistical package for the Social Sciences version 28.0 (SPSS, Chicago, IL, USA) for Windows. Distribution of data was verified by Kolmogorov–Smirnov test. Normally distributed data were expressed as mean (± s.d.) whereas not normally distributed data as median (interquartiles, IQR). Correlations were assessed using Spearman’s methods. Wilcoxon signed-rank test for non-normally distributed parameters or Student’s t-test for paired data for normally distributed parameters were used for comparisons among groups. A P-value of 0.05 was considered significant.

## Results

### Relationship between CellROX^®^ Orange positive spermatozoa and semen parameters

**Figure 2:**
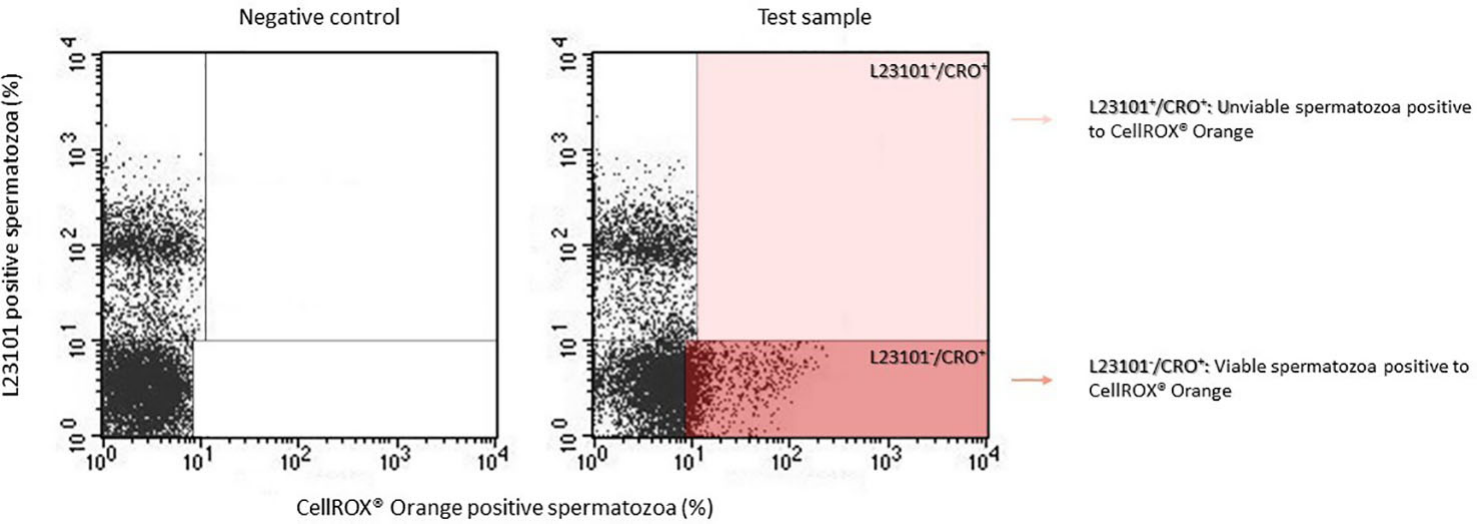
Typical dot plots of 4 similar experiments representing negative control (left) and test sample (right) related to CellROX^®^ Orange (red fluorescence) and L23101 (green fluorescence). CellROX^®^ Orange stains only L23101 negative events (dark red rectangle).

**Figure 3:**
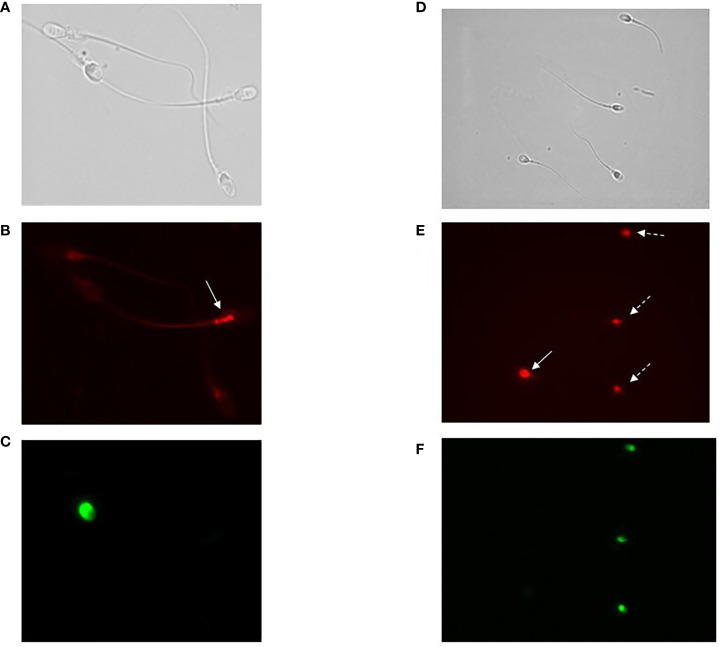
**(A–C)** Images of fluorescence microscopy of bright field **(A)**, CellROX^®^ Orange **(B)** and Y1 **(C)** stained spermatozoa. As observed, spermatozoa positive to CellROX^®^ Orange are negative to Y1 and vice versa. The arrow indicates the CellROX^®^ Orange fluorescent signal at midpiece level **(B)**. **(D–F)** Images of fluorescence microscopy of bright field **(D)**, DHE **(E)** and Y1 **(F)** stained spermatozoa. As observed, spermatozoa positive to DHE resulted to be both negative (white whole arrow) and positive (white dotted arrows) to Y1. The fluorescent signal related to the DHE is localized in the sperm head **(E)**.

Although the results of co-staining between CellROX^®^ Orange and Y1 (**Figure 1**) clearly show that CellROX^®^ Orange stains only Y1 negative and thus viable spermatozoa, we further verified this result by performing a double staining with Live/Dead fixable green stain L23101 (a probe that stably labels unviable spermatozoa) and CellROX^®^ Orange. As observed in **Figure 2**, CellROX^®^ Orange labeled only L23101 negative events (viable spermatozoa in lower right quadrant), whereas unviable cells (L23101 positive events) did not stain with CellROX^®^ Orange (upper right quadrant). CellROX^®^ Orange fluorescent signal is localized in the midpiece (**Figure 3A, B**), in agreement with a previous study (12). Spermatozoa labeled with CellROX^®^ Orange did not show the Y1 green fluorescence (**Figures 3B, C**).

**Figure 4:**
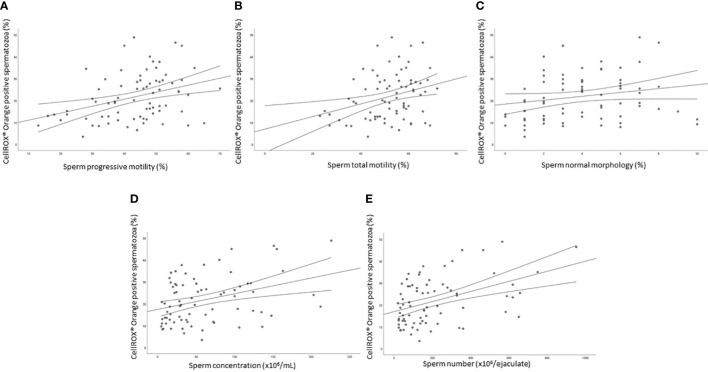
Scatter plots representing the relationships between the percentage of CellROX^®^ Orange positive spermatozoa and progressive (**A**, R=0.4, p<0.01) and total (**B**, R=0.3, p<0.05) motility, normal morphology (**C**, R=0.2, p<0.05), concentration (**D**, R=0.3, p<0.01) and number (**E**, R=0.4, p<0.01) in 77 subjects.

The median value of the percentage of viable CellROX^®^ Orange positive spermatozoa evaluated in 77 subjects was 20.1% [IQR: 12.7-29.1]. A statistically significant positive relationship was observed between the percentage of CellROX^®^ Orange positive spermatozoa and progressive (R=0.4, p<0.01, n=77; **Figure 4A**) and total (R=0.3, p<0.05, n=77; **Figure 4B**) motility, normal morphology (R=0.2, p<0.05, n=77; **Figure 4C**), concentration (R=0.3, p<0.01, n=77; **Figure 4D**) and number (R=0.4, p<0.01, n=77; **Figure 4E**). When subjects were divided into two groups according to semen characteristics (normozoospermic vs. non-normozoospermic, the latter showing at least one parameter below the 5th percentile of reference values of WHO semen manual (2010)), no statistically significant difference in CellROX^®^ positivity was found between the two groups (24.7 [IQR: 15.3-29.2], n=40 in normozoospermic vs 16.3 [IQR: 11.3-28.1], n=37 in non-normozoospermic men).

### Relationship between CellROX^®^ Orange positivity and sperm DNA fragmentation

**Figure 5:**
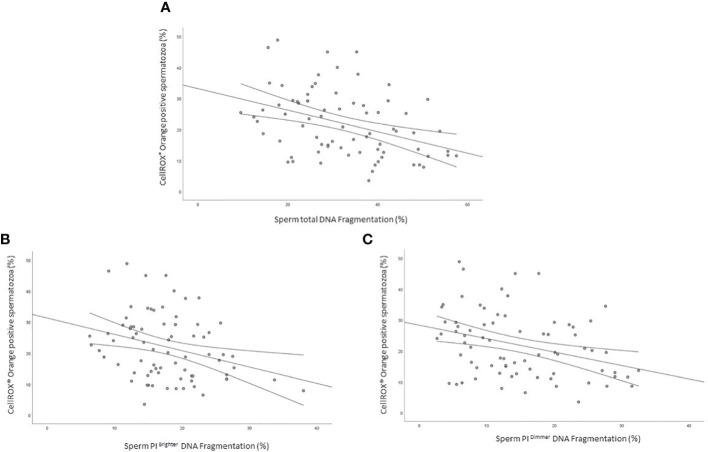
Scatter plots representing the relationship between the percentage of CellROX^®^ Orange positive spermatozoa and total (**A**, R=-0.4, p<0.01), PI Brighter (**B**, R=-0.3, p<0.05) and PI Dimmer (**C**, R=-0.3, p<0.01) DNA fragmentation in 76 subjects.

As shown in **Figure 5** the percentage of CellROX^®^ Orange positive spermatozoa was negatively correlated with sDF both in the total (R=-0.4, p<0.01, n=76; **Figure 5A**), PI brighter (R=-0.3, p<0.05, n=76; **Figure 5B**) and PI dimmer (R=-0.3, p<0.01, n=76; **Figure 5C**) sperm populations (14).

### Relationship between DHE positivity and semen parameters

**Figure 6:**
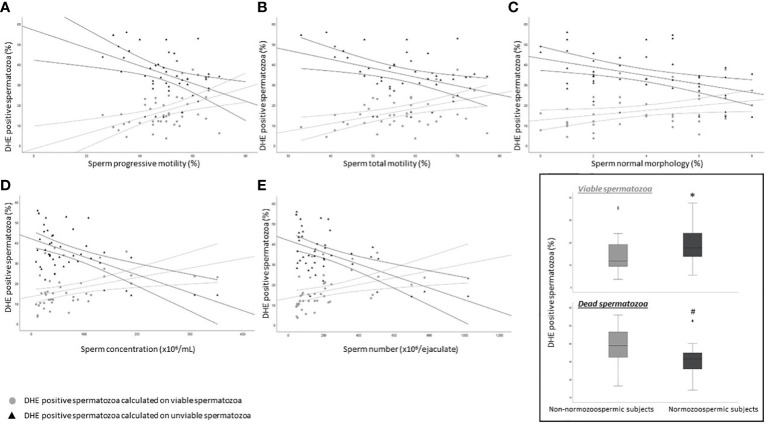
Scatter plots representing the relationship between the percentage of DHE viable (grey circles) and unviable (black triangles) positive spermatozoa and progressive (**A**, viable: R=0.5, p<0.01; unviable: R=-0.4, p<0.01) and total (**B**, viable: R=0.5, p<0.01; unviable: R=-0.4, p<0.01) motility, normal morphology (**C**, viable: R=0.4, p<0.05; unviable: R=-0.4, p<0.05), concentration (**D**, viable: R=0.5, p<0.01; unviable: R=-0.5, p<0.01) and number (**E**, viable: R=0.6, p<0.01; unviable: R=-0.4, p<0.01) in 44 subjects. Inset: box plots of the percentage of DHE viable (upper panel) and unviable (lower panel) positive spermatozoa in Non-normozoospermic (n=24) and Normozoospermic subjects (n=20). *p<0.05 vs. Non-normozoospermic subjects; #p<0.01 vs. Non-normozoospermic subjects.

Our results suggest that oxidized viable spermatozoa detected by CellROX^®^ Orange are related to a better semen quality. According to Escada-Rebelo et al. (12), CellROX^®^ Orange reagent reveals H_2_O_2_ but is not specific toward O2^•-^, whereas DHE shows distinct specificity toward both superoxide anion and hydrogen peroxide. Moreover, DHE, unlike CellROX^®^ Orange, reveals ROS both in viable and unviable cells. When the relationship between the percentage of DHE positive cells and semen parameters was evaluated, we found two opposite trends. DHE positive/Y1 negative spermatozoa, were significantly positively associated with progressive (R=0.5, p<0.01, n=44, **Figure 6A** grey circles) and total (R=0.5, p<0.01, n=44, **Figure 6B** grey circles) motility, normal morphology (R=0.4, p<0.05, n=44, **Figure 6C** grey circles), sperm concentration (R=0.5, p<0.01, n=44, **Figure 6D** grey circles) and number (R=0.6, p<0.01, n=44, **Figure 6E** grey circles). Conversely, when we considered DHE positive/Y1 positive (unviable) spermatozoa, significant negative correlations with all semen parameters were found (progressive motility: R=-0.4, p<0.01, n=44, **Figure 6A** black triangles; total motility: R=-0.4, p<0.01, n=44, **Figure 6B** black triangles; normal morphology: R=-0.4, p<0.05, n=44, **Figure 6C** black triangles; concentration: R=-0.5, p<0.01, n=44, **Figure 6D** black triangles; number: R=-0.4, p<0.01, n=44, **Figure 6E** black triangles). Furthermore, whereas the percentage of viable DHE positive spermatozoa was higher in normozoospermic subjects respect to non-normozoospermic ones (17.6% [IQR: 13.5-24.3], n=20 vs. 11.7 [IQR: 9.2-19.6], n=24, p<0.05), the percentage of DHE positive unviable spermatozoa was significantly lower in the former (31.4% [IQR: 25.2-35.0], n=20 vs. 38.8 [IQR: 32.1-46.5], n=24, p<0.01) (**Figure 6** insert). The median values of DHE positivity in viable and unviable spermatozoa in the 44 subjects were 15.0% [IQR: 10.7-23.3] and 34.2% [IQR: 29.3-43.6] respectively. DHE positive signal on spermatozoa is localized in the head (**Figures 3D, E**) of both viable (negative to Y1, **Figure 3F**) and unviable (positive to Y1, **Figure 3F**) spermatozoa, in agreement with a previous paper (12).

### Relationship between DHE positivity and sperm DNA fragmentation

**Figure 7:**
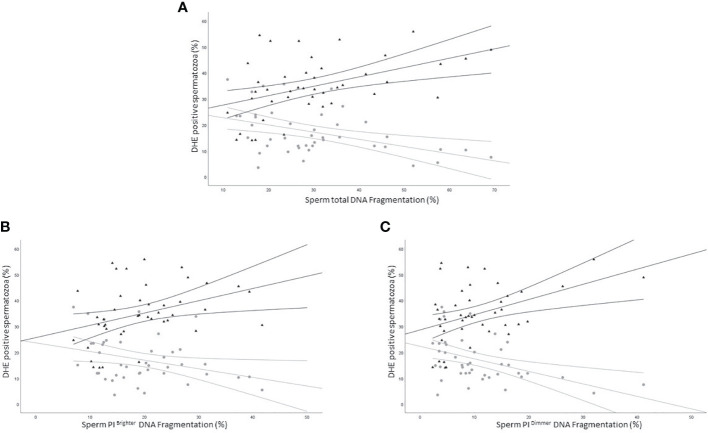
Scatter plots representing the relationship between the percentage of DHE viable (grey circles) and unviable (black triangles) positive spermatozoa and total (**A**, viable: R=-0.4, p<0.05; unviable: R=0.5, p<0.01), PI brighter (**B**, viable: R=-0.3, p=ns; unviable: R=0.4, p<0.01) and PI dimmer (**C**, viable: R=-0.4, p<0.01; unviable: R=0.3, p<0.05) DNA fragmentation in 43 subjects.

We next evaluated the relationship between the percentage of DHE positive viable and unviable spermatozoa and sDF. Viable DHE positive spermatozoa were negatively correlated with DNA fragmentation in total (R=-0.4, p<0.05, n=44; **Figure 7A** grey circles), PI brighter (R=-0.3, p=ns, n=44; **Figure 7B** grey circles) and PI dimmer (R=-0.4, p<0.01, n=44; **Figure 7C** grey circles) sperm populations. Conversely, in dead cells, positive associations were observed with the three DNA fragmented sperm populations (total: R=0.5, p<0.01, n=44; **Figure 7A** black triangles; PI brighter: R=0.4, p<0.01, n=44; **Figure 7B** black triangles; PI dimmer: R=0.3, p<0.05, n=44; **Figure 7C** black triangles).

### Relationship between CellROX^®^ Orange positivity and apoptosis

**Figure 8:**
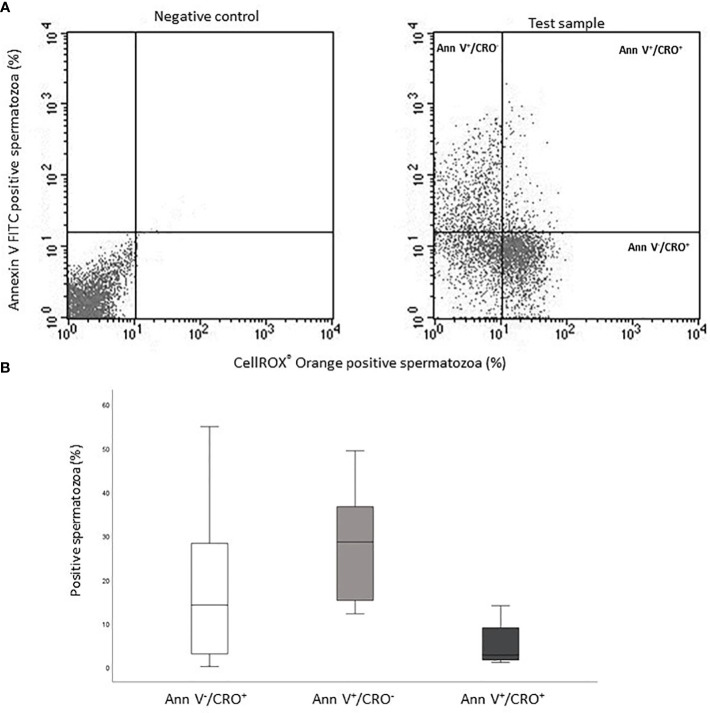
**(A)** Typical dot plots representing negative control (left) and test sample (right) related to CellROX^®^ Orange (red fluorescence, CRO) and Annexin V (green fluorescence, Ann V). **(B)** Box plots of the percentages of CellROX^®^ Orange positive and Annexin V negative spermatozoa (Ann V^-^/CRO^+^), CellROX^®^ Orange negative and Annexin V positive spermatozoa (Ann V^+^/CRO^-^) and CellROX^®^ Orange and Annexin V double positive spermatozoa (Ann V^+^/CRO^+^), n=12.

**Figure 9:**
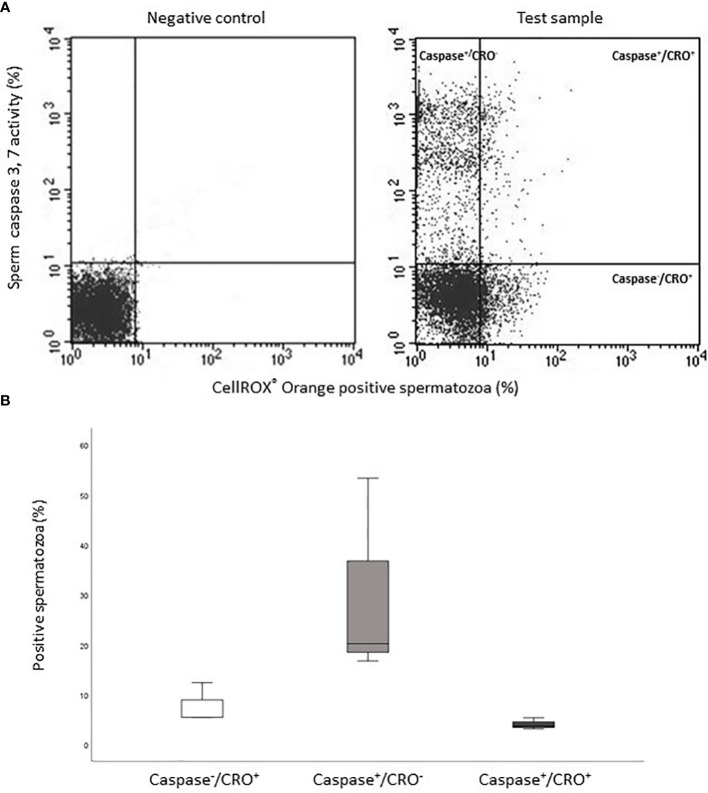
**(A)** Typical dot plots representing negative control (left) and test sample (right) related to CellROX^®^ Orange (red fluorescence, CRO) and Caspase 3,7 activity evaluated by FLICA (green fluorescence, Caspase). **(B)** Histograms of the percentages of CellROX^®^ Orange positive and Caspase 3, 7 activity negative spermatozoa (Caspase^-^/CRO^+^), CellROX^®^ Orange negative and Caspase 3, 7 activity positive spermatozoa (Caspase^+^/CRO^-^) and CellROX^®^ Orange and Caspase 3, 7 activity double positive spermatozoa (Caspase^+^/CRO^+^), n=3.

The occurrence of positive correlations with functional parameters, indicates that ROS revealed by CellROX^®^ Orange and DHE in viable spermatozoa are related to a better sperm quality. To further investigate this possibility, we double labeled spermatozoa with CellROX^®^ Orange and Annexin V, which detects phosphatidylserine exposure, representing an early sign of apoptosis (17, 18). In **Figure 8A**, the typical dot plots of the negative control and test sample of the two fluorescent probes are shown. Positive spermatozoa for both probes (oxidized and early apoptotic spermatozoa) are located in the upper right quadrant of the test sample dot plot (**Figure 8A**). As shown in **Figure 8B**, on average, only 2.8% [IQR 1.7-9.4] (n=12) of Annexin V positive spermatozoa was also positive for CellROX^®^ Orange. In addition, double staining with CellROX^®^ Orange and caspase 3, 7 activity (a late sign of apoptosis, detected by FLICA^TM^ kit) was performed. The typical dot plots of the negative control and test sample of CellROX^®^ Orange and FLICA are reported in **Figure 9A**. Only 4.1 ± 1.2% (n=3) of spermatozoa expressing caspase 3, 7 activity were also CellROX^®^ Orange positive (**Figure 9B**). These results indicate that CellROX^®^ Orange mostly identifies spermatozoa without apoptotic features.

### Oxidized viable spermatozoa in swim-up selected samples

**Figure 10:**
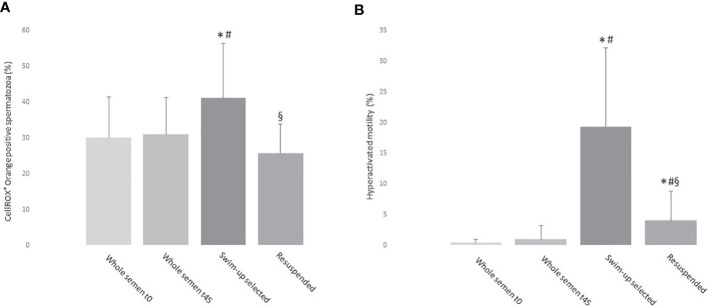
Histograms representing the mean values ( ± sd) of the percentages of CellROX^®^ Orange positive spermatozoa (**A**, n=11) and hyperactivated motility (**B**, n=11) of whole semen at time zero (t0) and after 45 minutes at 37°C (t45), swim-up selected samples and swim-up selected spermatozoa then resuspended with the lower phase. *p<0.05 vs. whole semen t0; ^#^p<0.05 vs. whole semen t45; ^§^p<0.05 vs. swim-up selected sample.

Next, we evaluated CellROX^®^ positivity after swim-up selection, a procedure used to prepare spermatozoa for assisted reproduction. To this aim, CellROX^®^ positivity was evaluated in whole semen samples at the beginning (t0) and end (t45) of incubation for swim-up selection (to verify that eventual differences in the percentage of CellROX^®^ positivity in selected samples was not due to the incubation time), in swim-up selected sperm, and in resuspended selected spermatozoa [to verify that eventual changes of CellROX^®^ positivity in selected samples did not depend on selection technique or incubation time, both being reported as possible causes of ROS increase (19)]. In **Figure 10A**, the mean percentage of CellROX^®^ Orange positive spermatozoa for each experimental point is reported. No statistically significant differences were found between whole semen samples at t0 (30.1 ± 11.4%, n=11) and at t45 (31.0 ± 10.3%, n=11). A statistically significant increase in the percentage of CellROX^®^ positive spermatozoa was observed in selected samples respect to whole semen at both time points. Such an increase was not detected in resuspended samples. Kinematic sperm parameters, evaluated by CASA analysis in the four experimental points, were significantly improved in swim-up selected samples respect to whole semen at t0 and t45 (**Table 2**). Resuspended samples showed an intermediate pattern of kinematic parameters respect to both whole semen and selected samples (**Table 2**). As expected, hyperactivated motility (**Figure 10B**) was significantly increased in swim-up respect to resuspended and whole semen samples (t0 and t45).

**Table 2:** Mean ± sd of kinematic sperm parameters, evaluated by CASA system, are reported in whole semen t0, whole semen t45, swim-up selected and resuspended samples (n=11).

Sample	VAP (µm/s)	VSL (µm/s)	VCL (µm/s)	ALH (µm)	BCF (Hz)	LIN (%)	STR (%)
whole semen t0	25.7 ± 7.8	20.2 ± 6.0	40.3 ± 13.3	2.7 ± 0.9	18.8 ± 2.8	55.0 ± 8.5	81.2 ± 5.9
whole semen t45	28.3 ± 9.8	22.6 ± 7.4	43.8 ± 18.3	3.0 ± 1.3	21.4 ± 2.4	55.2 ± 9.5	81.1 ± 5.9
swim-up selected	62.3 ± 11.7^a,b^	46.9 ± 9.1^a,b^	109.0 ± 22.4^a,b^	6.6 ± 1.5^a,b^	18.8 ± 1.6^a,b^	48.1 ± 6.1^a,b^	77.4 ± 5.2
resuspended	33.8 ± 14.2^a,c^	27.9 ± 11.0^a,c^	54.4 ± 26.7^a,c^	3.3 ± 1.8^c^	22.6 ± 2.6^a,c^	60.6 ± 11.3^c^	85.7 ± 5.9^c^

^a^p<0.05 vs. whole semen t0; ^b^p<0.05 vs. whole semen t45; ^c^p<0.05 vs. swim-up selected sample.

### CellROX^®^ Orange and DHE positivity after induction of oxidative stress

**Figure 11:**
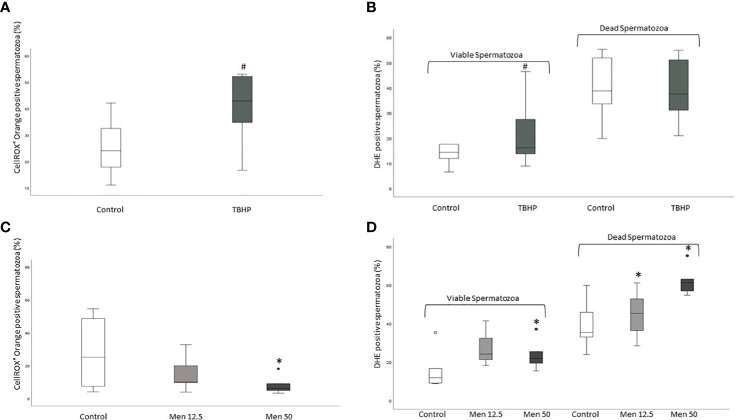
Box plots of the percentages of CellROX^®^ Orange (**A**, only viable spermatozoa) and DHE (**B**, viable and unviable spermatozoa) positive spermatozoa in control samples (incubated only with medium) and after incubation for 30 minutes with TBHP (1mM) and box plots of the percentages of CellROX^®^ Orange (**C**, only viable spermatozoa) and DHE positive spermatozoa (**D**, viable and unviable spermatozoa) in control samples (incubated only with medium) and after incubation for 30 minutes with Menadione (12.5 and 50 µM). ^#^p<0.01 vs. CTRL; *p<0.05 vs. Control.

To demonstrate that the two probes are sensitive to an induction of ROS in spermatozoa, we used Tert-Butyl hydroperoxide (TBHP, 1mM) and Menadione (12.5 and 50 µM). The first is a well-characterized cell membrane permeable pro-oxidant that generates free radical peroxides (20), whereas the second is a superoxide generator (21). After incubation with TBHP an increase of the percentage of positivity to both CellROX^®^ Orange (**Figure 11A**) and DHE in viable (but not unviable) spermatozoa (**Figure 11B**) was observed. When Menadione was added to spermatozoa, a noticeable increase in DHE positivity, both in viable and unviable spermatozoa (**Figure 11D**) was observed with both concentrations. Conversely, CellROX^®^ Orange positivity decreased after exposure to 50 µM Menadione (**Figure 11C**).

**Figure 12:**
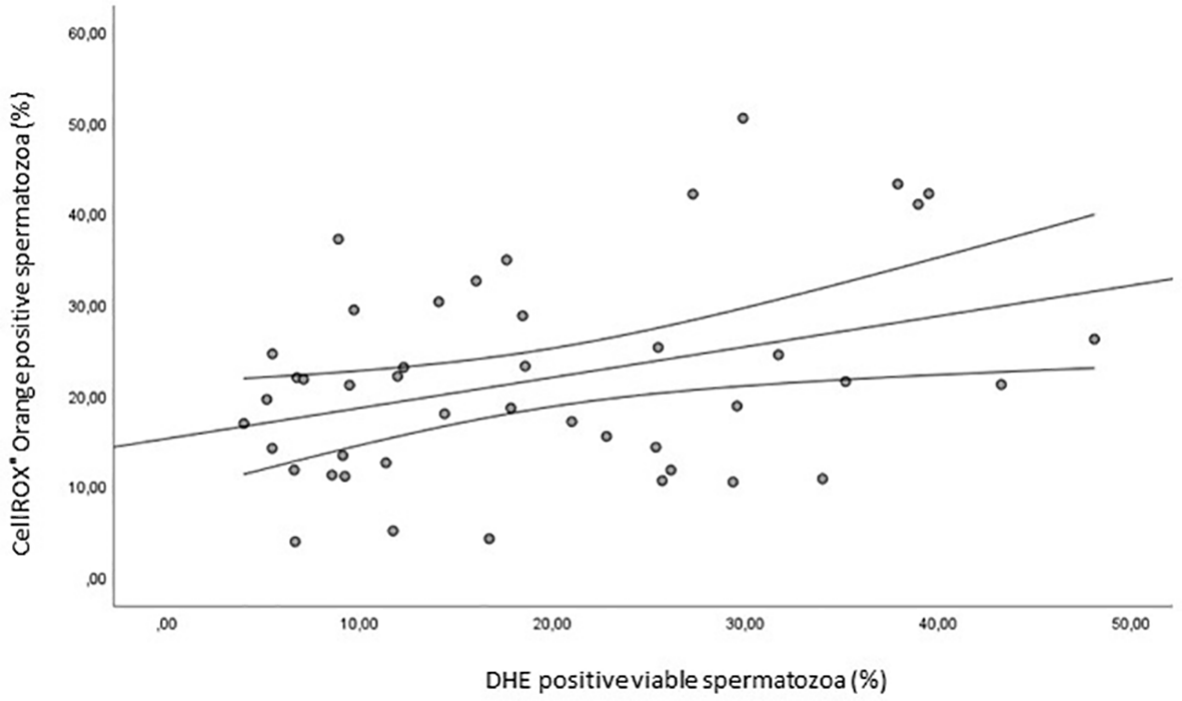
Scatter plot representing the relationships between the percentage of CellROX^®^ Orange positive spermatozoa and the percentage of DHE positive viable spermatozoa (R=0.4, p<0.05, n=44).

Finally, we evaluated the correlation between CellROX^®^ Orange and DHE ROS detection in viable spermatozoa. As shown in **Figure 12**, a positive correlation between the two probes was found (R= 0.4, p<0.05, n=44).

## Discussion

Oxidative stress in spermatozoa must be well balanced. Indeed, whereas low levels of ROS are required for the different sperm functions, mediating fundamental physiological mechanisms, high ROS levels may lead to clear negative effects including membrane damages and protein and DNA oxidation and fragmentation (22). At present, several methods have been devolved to measure free radicals in semen reporting a negative association between ROS and semen quality (23). However, none of these methods is actually used in clinical male infertility work up due to the lack of conclusive proofs of their diagnostic relevance.

The main novel finding of our study is the demonstration that the percentage of viable oxidized spermatozoa detected by CellROX^®^ Orange and DHE is positively related, albeit with moderate correlation coefficients, with concentration, number, motility and typical morphology and negatively related with sDF. The fact that such correlations are weak, suggests that within the viable oxidized spermatozoa detected with both probes there are cells with physiological and non-physiological intracellular levels of ROS, the former likely representing the majority and driving the positive correlations. Clearly, it would be of interest to develop tools able to evaluate at what levels ROS species cease to be functional and become deleterious for spermatozoa. We show here that only a small fraction of viable oxidized spermatozoa shows signs of apoptosis. Whether such spermatozoa express non-physiological levels of intracellular ROS remains to be determined.

Few studies investigated the association between intracellular oxidative stress and sperm parameters, showing different results depending on the probe used to evaluate ROS and on the analyzed sperm population (24, 25). In particular, when DHE was used without distinguishing between viable and unviable spermatozoa, a negative association with semen parameters was found (24). Conversely, Kiani-Esfahani et al. (25), by using DHR123 (a probe evaluating H_2_O_2_ produced by spermatozoa) coupled to PI to distinguish viable spermatozoa, found a positive association between oxidized viable spermatozoa and semen quality, in agreement with our results. In our study, the association between ROS in viable sperm and good semen quality is reinforced by the fact that the percentage of DHE positive viable cells is higher in the subjects classified as normozoospermic, i.e. with parameters of concentration, motility and normal morphology above the 5th percentile of WHO manual 2010 (13) reference values. As mentioned, we found a negative relationship between viable oxidized spermatozoa and sDF, suggesting that ROS levels detected with our probes are poorly involved in generating DNA breaks. Although such result appears in contrast with the current view that oxidative stress is one of the mechanisms leading to sperm DNA damage (26), we should consider that a positive association was found when oxidized unviable spermatozoa were considered, in agreement with other studies (27). To our knowledge, only one study evaluated the relationship between the two parameters considering viable oxidized spermatozoa (28), reporting a positive association. In this study, however, sDF was evaluated by sperm chromatin dispersion test, which detects the susceptibility of sperm chromatin to damage rather than the occurrence of real DNA breaks as done by TUNEL (29). In addition, the positive correlation was assessed only in 28 men, selected on the basis of unexplained infertility (28). Such differences might explain the different results respect to our study.

As mentioned, the two probes used in our study reveal different ROS species: CellROX^®^ Orange detects hydrogen peroxide, whereas DHE both hydrogen peroxide and superoxide anion (12). The specificity of the two probes towards the different ROS was here confirmed by incubating spermatozoa with an analog of H_2_O_2_ (TBHP) and Menadione which generates superoxide. The occurrence of a positive correlation between the two probes in viable spermatozoa (**Figure 12**) was expected as both probes detect hydrogen peroxide. The fact that the correlation between the two probes is not so strong, might be due to the fact that DHE detects also superoxide anion.

To understand better the relationship between oxidative stress and sperm functions, we evaluated the oxidative status after swim-up, an *in vitro* procedure that mimics sperm selection occurring in the female genital tract and that is used in assisted reproduction (19). Surprisingly, we found a higher percentage of CellROX^®^ Orange positive spermatozoa after swim-up. Selected sperm with such procedure are characterized by better motility [including hyperactivation, and other kinematic parameters (30)], morphology and lower sDF (31). The increase in ROS positive spermatozoa does not appear to be due to the technique or the incubation time, both reported as possible causes of ROS generation (19), because the aliquots of swim up semen samples resuspended by mixing the upper and the lower phases showed a percentage of oxidized viable spermatozoa similar to whole semen at t0 and t45. Such finding indicates that a fraction of ROS positive spermatozoa are selected from raw semen during swim-up. In particular, in our experimental setting, up to 40% of selected spermatozoa are ROS positive. Clearly, studies investigating the association with assisted reproduction outcomes will be necessary to confirm that ROS detected in viable spermatozoa in raw semen or after swim-up by these two probes are a marker of good sperm performance.

It must be considered that a simple statistical relationship between two parameters cannot answer to the question of whether the two parameters do or do not co-exist in the same cell. By evaluating the co-staining of CellROX^®^ Orange and Annexin V [early signs of apoptosis (18)] and CellROX^®^ Orange and caspase 3, 7 activity [late signs of apoptosis (32)] we demonstrated that most oxidized viable spermatozoa do not show apoptotic features. Such result might also explain the negative relationships found with sDF, as apoptosis is one of the main mechanisms leading to DNA breaks in spermatozoa (26).

In conclusion, our study demonstrates that evaluation of hydrogen peroxide and superoxide anion in viable spermatozoa with CellROX^®^ Orange and DHE, allows identification of the oxidized semen sperm fraction related to better performances. The two probes may be useful to determine such fraction likely improving the diagnostic process of male infertility.

## Data availability statement

The raw data supporting the conclusions of this article will be made available by the authors, without undue reservation.

## Ethics statement

The studies involving human participants were reviewed and approved by Comitato Etico Area Vasta Centro at Azienda Ospedaliero Universitaria Careggi *via* Largo Brambilla 3, 50134 Firenze. The patients/participants provided their written informed consent to participate in this study.

## Author contributions

Conceptualization, EB and SM; Methodology, LT; Validation, GT, LT, and SM; Statistical Analysis, SM; Investigation, GT; Data Curation, LT; Writing – Original Draft Preparation, SM; Writing – Review & Editing, EB and LV; Supervision, LV; Funding Acquisition, EB. All authors contributed to the article and approved the submitted version.

## Funding

This research was funded by the Italian Ministry of Education, University and Research (PRIN 2017 project to EB, Grant Number 2017TK7Z8L_006) and University of Florence.

## Acknowledgments

We thank Drs. Sara Dabizzi, Selene Degl’Innocenti and Maria Grazia Fino for semen sample evaluation.

## Conflict of interest

Dr. SM declares to be Associate Editor of Frontiers in Endocrinology, Section: Reproduction. Dr. LT declares to be Topic Editor for the research topic “Women in Reproductive Endocrinology 2022”.

The remaining authors declare that the research was conducted in the absence of any commercial or financial relationships that could be construed as a potential conflict of interest.

## Publisher’s note

All claims expressed in this article are solely those of the authors and do not necessarily represent those of their affiliated organizations, or those of the publisher, the editors and the reviewers. Any product that may be evaluated in this article, or claim that may be made by its manufacturer, is not guaranteed or endorsed by the publisher.

## Abbreviations

ALH, amplitude of lateral head displacement. BCF, beat cross frequency. C.A.S.A., Computer-Assisted-Sperm Analysis. DHE, dihydroethidium. HA, hyperactivated spermatozoa. HSA, human serum albumin. HTF, Human tubal fluid. LIN, linearity of progression. sDF, sperm DNA fragmentation. STR, straightness. TBHP, Tert-Butyl hydroperoxide. VAP, Average path velocity. VCL, curvilinear velocity. VSL, straight line velocity.
